# Spatial-Temporal Variations of Chlorophyll-a in the Adjacent Sea Area of the Yangtze River Estuary Influenced by Yangtze River Discharge

**DOI:** 10.3390/ijerph120505420

**Published:** 2015-05-20

**Authors:** Ying Wang, Hong Jiang, Jiaxin Jin, Xiuying Zhang, Xuehe Lu, Yueqi Wang

**Affiliations:** 1 Jiangsu Provincial Key Laboratory of Geographic Information Science and Technology, Nanjing University, Xianlin Road 163, Nanjing 210023, China; E-Mails: mfacewang@gmail.com (Y.W.); jiaxinking@126.com (J.J.); lzhxy77@gmail.com (X.Z.); luxh43@gmail.com (X.L.); 2 International Institute for Earth System Science, Nanjing University, Xianlin Road 163, Nanjing 210023, China; 3 Key Laboratory of Coastal Zone Environmental Processes and Ecological Remediation, Yantai Institute of Coastal Zone Research, Chinese Academy of Sciences, Yantai 264003, China; E-Mail: yuwyuw1306@126.com

**Keywords:** spatial-temporal variations, chlorophyll-a, discharge, Yangtze River estuary, SeaWiFS

## Abstract

Carrying abundant nutrition, terrigenous freshwater has a great impact on the spatial and temporal heterogeneity of phytoplankton in coastal waters. The present study analyzed the spatial-temporal variations of Chlorophyll-a (Chl-a) concentration under the influence of discharge from the Yangtze River, based on remotely sensed Chl-a concentrations. The study area was initially zoned to quantitatively investigate the spatial variation patterns of Chl-a. Then, the temporal variation of Chl-a in each zone was simulated by a sinusoidal curve model. The results showed that in the inshore waters, the terrigenous discharge was the predominant driving force determining the pattern of Chl-a, which brings the risk of red tide disasters; while in the open sea areas, Chl-a was mainly affected by meteorological factors. Furthermore, a diversity of spatial and temporal variations of Chl-a existed based on the degree of influences from discharge. The diluted water extended from inshore to the east of Jeju Island. This process affected the Chl-a concentration flowing through the area, and had a potential impact on the marine environment. The Chl-a from September to November showed an obvious response to the discharge from July to September with a lag of 1 to 2 months.

## 1. Introduction

Phytoplankton is one of the major factors in the carbon cycle in the ocean-atmospheric system and climate change [1], and it is also an important indicator of marine primary productivity, eutrophication status and fishery resources [2]. The two dominant factors influencing phytoplankton growth are light and nutrients, although temperature can affect phytoplankton growth [3]. River discharges consist of numerous nutrients (e.g. phosphate, nitrate) which are one of the dominant factors affecting phytoplankton in coastal waters, especially nearly estuaries [4]. The concentrations of these nutrients are closely related to terrestrial ecosystem processes and human activities. Therefore, the influence of terrestrial discharge on coastal phytoplankton could reflect the impact of terrestrial ecological processes on the marine environment, which is closely related to human health and the living environment. Chlorophyll-a (Chl-a) can be considered a biomarker of aquatic ecosystems [5]. Therefore, Chl-a concentrations have a guiding effect in the fields correlated with environmental protection and human health, as in the cases of red tide disaster prevention, marine environmental protection, mechanisms of formation of fishing grounds and so on [6]. 

Satellite-based remote sensing Chl-a concentration data derived from ocean color sensors such as Coastal Zone Color Scanner (CZCS), Moderate Resolution Imaging Spectroradiometer (MODIS), Sea-viewing Wide Field-of-view Sensor (SeaWiFS), have been widely used in recent ocean environment monitoring studies. Particularly, the Chl-a concentration derived from SeaWiFS provides a valuable dataset for long-term studies. A match up analysis comparing SeaWiFS-derived and *in situ*-measured Chl-a concentrations from about 0.02‒7 mg·m^−3^ was carried out by Bailey *et al.* [7], who highlighted the reasonably high quality of the Chl-a data derived from the ocean color 4-band algorithm (OC4) around the world. Gregg *et al.* [8] evaluated the SeaWiFS Chl-a dataset at the global and regional scales, proving that SeaWiFS data in the entire Pacific Ocean exhibited a very good correspondence with *in situ* data. Also, based on the comparison of the RMS log errors on a global and coastal scale, the global error was 31% and the coastal was 33%. In conclusion, the studies confirmed that the SeaWiFS-derived Chl-a concentrations could represent the variations of Chl-a concentration in the Western Pacific coastal area. 

Generally, river plumes are turbid and rich in nutrients, remaining near the surface due to their buoyancy and breaking up into lenses of less saline water, stimulating phytoplankton growth [9]. Accordingly, several studies have investigated the influence of river discharges on primary production and Chl-a concentration distribution, in different estuary regions over the world like the northwestern coast of the Iberian Peninsula [5], the Pacific Northwest Coast [10], the northeastern Gulf of Mexico [11], and the coast off northern Japan [12]. Most of these studies were based on Chl-a remote sensing imagery data complemented by *in situ* measurements to describe and analyze the spatial variation of phytoplankton blooms promoted by discharge, and they concluded that the discharge increased the Chl-a concentration in the estuaries.

The Yangtze River, the longest river in Asia, flows through plateaus and plains with large segments of natural and anthropogenic sources [13]. Diluted water from the Yangtze River spreads eastwards and over a broad area of the East China Sea, reaching as far as Jeju Island to the Tsushima Strait [14,15,16]. The East China Sea, where the Zhoushan fishing ground is located, is rich in fishery resources. Meanwhile, the East China Sea is a frequent harmful algal bloom area. These frequent harmful algal blooms destroy the marine ecological environment, and impact the food safety of marine fishes [6]. It is, therefore, a typical coastal area to study the impact of terrigenous discharges on the Chl-a concentration in an inshore estuary.

Previous coupled studies have revealed that how discharges influence the Chl-a concentration in the sea area along the Yangtze River [17,18,19,20]. Chen *et al*. [21] used the SeaWiFS data with the OC4 algorithm, which was developed using a large (*N* = 2853) *in situ* dataset [22], to study the spring blooms in the Yangtze River Estuary. They discussed the uncertainty of the classification between Case 1 and Case 2 waters, which were defined by Morel and Prieur [23], and verified the accuracy of remote sensing data with the *in situ* data. Kim *et al*. [24] concluded that the spatial distributions of the diluted water area, reflected by the high Chl-a concentration, correlated with the inter-annual variation in the summer freshwater discharge, and the diluted water spread eastward in the East China sea with a time lag of 1 to 2 months after the discharge. However, few studies have been conducted on the quantitative influence of river discharges on Chl-a concentrations on the temporal and spatial scale. The quantitative relationship between the river discharge and Chl-a concentration is an effective means to explore the responses of coastal marine environments to terrestrial human activities and to provide a scientific basis for governments to enact policies to protect the coastal marine environment.

The present work aims to analyze the influence of the Yangtze River discharge on Chl-a concentrations, including the influenced spatial regions and the seasonal variation, and also the impact on open sea areas. Firstly the spatial-temporal variations of Chl-a concentration are explored combined with the influence of the Yangtze River discharge. Then the sinusoidal model is used to determine whether the Chl-a is gradually affected by the seasonal variation in discharge from inshore to open sea. Finally, the extension process of the diluted water from the Yangtze River and the temporal variations in the influence of discharge on Chl-a concentration are investigated.

## 2. Materials and Methods

### 2.1. Study Area

**Figure 1:**
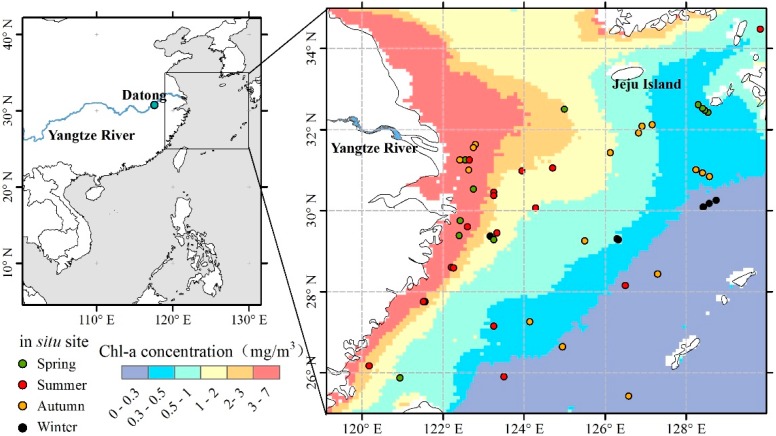
Study area and the average annual Chl-a concentration from 1998 to 2010 obtained from Sea-viewing Wide Field-of-view Sensor (SeaWiFS) data. The point in the left picture indicates the location of the Datong hydrological gauging station. The points in different colors in the right picture indicate the sites of *in situ* Chl-a concentration measurements for four seasons.

The study area is located at 25° N ~ 35° N, 120° E ~ 130° E, with the Yangtze River estuary at the center, including the north part of the East China Sea and the Yellow Sea (Figure 1). The Yangtze River is the third longest river in the world. On average it carries about 433 million tons of sediment and 150 million tons of dissolved matter to the East China Sea every year [13]. These terrigenous materials provide rich substrates for biological activity in the ocean [13]. This distinctive area is located in the west coast of the Pacific, with a typical monsoon climate. Therefore, the study of coastal waters from the influence of continental water to coastal ecological environments is significant.

### 2.2. Dataset

The monthly averaged Chl-a concentrations from September 1997 to December 2010 were acquired from Level 3 Standard Mapped Images of SeaWiFS derived from the ocean color 4-band algorithm (OC4) [25]. To verify the accuracy of the Chl-a remote sensing data, 58 samples of *in situ* Chl-a concentration data were collected from the studies of published papers [21,26,27], the SeaWiFS Bio-optical Archive and Storage System (SeaBASS) [28] and the National Oceanographic Data Center (NODC) [29]. The *in situ* data were obtained by survey cruises and dip samples taken from 0–5 m depth and analyzed by high-performance liquid chromatography (HPLC) to obtain the phytoplankton Chl-a concentration. Uncertainty of *in situ* Chl-a concentration exists due to the limitations of the samples, but HPLC is generally regarded as a precise method for Chl-a measurement and provides reliable data to verify the satellite data. All of the *in situ* data were scattered over the research area as shown in Figure 1. The survey cruises were carried out between November 1997 and February 2006. Therefore, it is representative as validation data.

The monthly mean of the Yangtze River discharges from 1998 to 2007 were observed at the Datong hydrological gauging station (117°37′ E, 30°46′ N) [24], which is shown in Figure 1. The hydrological station measured the river discharge in real-time by a current meter, and obtained a discharge value once every hour. The monthly discharge figures are the average value for each month. The Datong station, located in the estuary tidal limit, is the last downstream station of the Yangtze River with a distance of approximately 680 km to the river mouth [17]. The runoff observed at Datong Station represents the Yangtze River discharge.

### 2.3. Method

#### 2.3.1. Method of Data Analysis

The SeaWiFS remote sensing data are reconstructed by the Data Interpolating Empirical Orthogonal Functions (DINEOF) [30,31] method to fill in missing data. A linear correlation analysis was conducted between the discharge and Chl-a concentration during the period from January 1998 to December 2007 to study the relationship between Chl-a concentration and discharge from the Yangtze River in the study area. For each pixel, the correlation between discharge and Chl-a concentration is analyzed. The study area was separated into five zones based on the diversity of correlation coefficients. 

A sinusoidal curve model is used to analyze the time series of Chl-a concentration and discharge from the Yangtze River. The magnitude, growth rate, amplitude, change cycle and other factors were analyzed after the sinusoidal curve model. The correlation coefficients between the monthly discharge from the Yangtze River and Chl-a around Jeju Island are calculated. The correlation coefficients in each pixel are calculated with a time lag of 0, 1, or 2 months to describe the progress of discharge extension.

#### 2.3.2. Data Reconstruction

**Figure 2:**
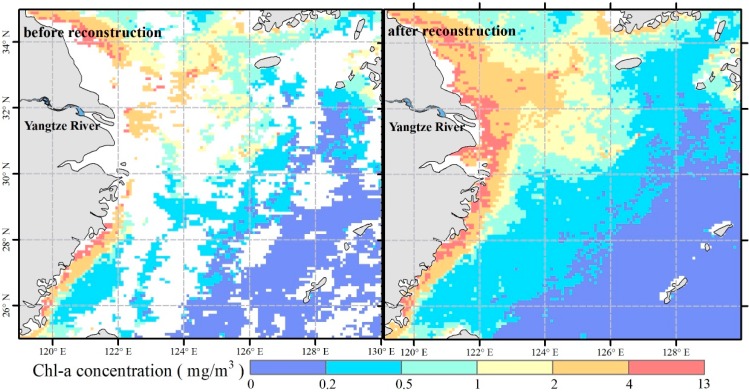
Chl-a concentrations from remote sensing technique before and after reconstruction by Data Interpolating Empirical Orthogonal Functions (DINEOF). Note the image from September 1997 for example.

Due to clouds, the retrieved Chl-a concentration maps have much missing data. To reconstruct the missing data, the DINEOF method [30], which was designed to fill the missing data in SST images [31] is proposed, and now has been applied to Chl-a maps [32,33]. DINEOF instead of using the direct minimization of expected error covariance as the objective of the interpolation, is a parameter-free (*i.e.*, the necessary parameters are derived internally from existing data), Empirical Orthogonal Functions-based method for reconstructing missing data. It is used for time series of images to provide a means to calculate the principal components of incomplete data as eigenvectors of a covariance matrix, while simultaneously filling in the missing data. Figure 2 shows remotely sensed data before and after DINEOF reconstruction. The data before reconstruction has large tracts of null values, which seriously limit data analysis. DINEOF reconstructs the null values by referring to the temporal and spatial relationships of surrounding data. Pixels and images with extremely high missing data coverage (>95%) are eliminated [33]. Furthermore, the DINEOF retains the original information and avoids value changes during processing.

#### 2.3.3. Sinusoidal Curve Model for Trend Analysis

In this study, a sinusoidal curve model is used to quantify the temporal variation characteristics of Chl-a concentration, comprising seasonal variation and linear trends [34]. This model was programmed in MATLAB to fit time series and simulate the change trends, variation periods and fluctuation ranges of Chl-a concentration from January 1998 to December 2010. The model is shown as Equation (1):

(1) $$Y_{t}=A+B\times X_{t}+C\times \sin(2\frac{\text{π}}{D}X_{t}+E)+N_{t}$$

where $Y_{t}$ is the mean monthly Chl-a concentration for each zone calculated by the model. $X_{t}$ is the time in months after January 1998 ($X_{t}$: 1 to 156 is equivalent to January 1998 to December 2010 for Chl-a concentration), A, B, C, D and E are the model parameters. $A+B\times X_{t}$ is the trend of variation of Chl-a concentration (A reflects the overall level of Chl-a concentration in each zone, B quantitatively compares the monthly variation trend of Chl-a concentration in different zones). B/A indicates the change rate of Chl-a concentration in each zone. $C\times \sin(2\frac{\pi }{D}X_{t}+E)$ represents the seasonal variation of Chl-a concentration (where C is the amplitude of variation, D is the change cycle, and E is the initial phase showing the floating characteristics with time).

The sinusoidal curve model is also applied in the discharge from the Yangtze River data with the same model and coefficients shown in Equation (1). The time span is from January 1998 to December 2007.

$N_{t}$ represents the residual error between the actual values and simulated values. Weatherhead *et al.* [34] assumed $N_{t}$ as autoregressive and can be described as follows:

(2) $$N_{t}=\Phi N_{t-1}+\varepsilon_{t}$$

where Φ is the autocorrelation among residual errors, and $\varepsilon_{t}$ is the autocorrelation noise. The autocorrelation among the residuals will influence the precision of Chl-a concentration variation trend. The precision of the model-simulated trend ($\sigma_{B}$) is a function of the autocorrelation (Φ), time span (*T*) and standard deviation of residuals ($\sigma_{N}$), and can be approximately represented as [34]:

(3) $$\sigma_{B}\approx \frac{\sigma_{N}}{T^{3/2}}\sqrt{\frac{1+\Phi }{1-\Phi }}$$

The computationalformula of the standard deviation is:

(4) $$\sigma_{N}=\sqrt{\frac{\sum_{j\;=\;1}^{T}\left(Y_{j}-\bar{Y}\right)}{T}}$$

If trend B and precision $\sigma_{B}$ meet the condition: $\left|\frac{B}{\sigma_{B}}\right|>2$, the variation trend (B) of the monthly average Chl-a concentration is considered significant when the confidencelevel is 95%.

The initial phase (E) indicates the positional relation between the sine function wave pattern and *X*-axis or the distance of lateral movement from the *X*-axis of the sine function we pattern. It shows the characteristic pattern of seasonal variation, which means that the curve riseand falls to reflect the high and low values. The distance of lateral movement from the *X*-axis (Dis) is calculated as:

(5) $$\text{Dis}=E\times \frac{D}{2\pi }$$

The time difference of wave pattern between discharge and each zone (Differ) is calculated as:

(6) $$\text{Differ}=\text{Dis}\left(\text{Zone i}\right)-\text{Dis}\left(\text{discharge}\right)=\left(E\left(\text{Zone i}\right)-E\left(\text{discharge}\right)\right)\times \frac{D}{2\text{π}}$$

where i refers to 1 to 5, and the units of Dis and Differ are a month.

## 3. Results and Discussion

### 3.1. Accuracy Assessment of the Remote Sensing Data

The 8-day averaged merged SeaWiFS remote sensing data is compared to *in situ* data. The same 8 days are set for the SeaWiFS data as the time window for matchup analysis, and the space window is a 9 km × 9 km grid (a single SeaWiFS pixel).

**Figure 3:**
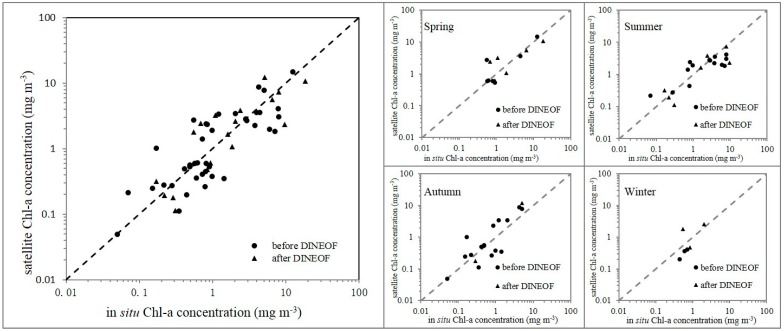
Comparison of the *in situ* Chl-a concentration with SeaWiFS remote sensing data for the entire time frame and for four seasons. The dots indicate the matchup between *in situ* data and satellite data before DINEOF interpolation, and the triangles indicate the matchup with the reconstructed data after DINEOF interpolation.

Figure 3 and Table 1 show a significant correlation between the satellite-based and *in situ* Chl-a concentration determination coefficient (*R*^2^) = 0.57, *p* < 0.0001, root mean square error (RMSE) = 2.32 mg/m^3^. The result of the linear regression is consistent with the research of Cui *et al.* [35]. Their published *in situ* dataset are the most comprehensive, high quality bio-optical oervation results along the China coast [36]. Therefore, it supports our research result as *in situ* data. The matchup result after DINEOF is consistent with the one before DINEOF, so the use of DINEOF is reasonable to reconstruct the remote sensing Chl-a concentration. Similar results were also obtained by Wang *et al*. [33].

Taking into account the seasonal variation of Chl-a, satellite data and *in situ* data were compared in each season respectively (Figure 3 and Table 1). The accuracy is high in spring (*R*^2^ = 0.81, *p* < 0.0001, RMSE = 2.44 mg/m^3^) and autumn (*R*^2^ = 0.90, *p* < 0.0001, RMSE = 2.23 mg/m^3^), but relatively low in summer (*R*^2^ = 0.43, *p* = 0.001, RMSE = 2.62 mg/m^3^) and winter (*R*^2^ = 0.59, *p* = 0.074, RMSE = 0.61 mg/m^3^). Generally, the correlation for each season is statistically significant except for winter where there were insufficient samples.

The agreement with the ground reference data showed that the reconstructed SeaWiFS remote sensing data could be used in further study to discuss the spatial and temporal variations of Chl-a in the study area although there are still some uncertainties for the Chl-a values retrieved from satellite-based data.

**Table 1:** Comparison of the *in situ* Chl-a concentration with reconstructed SeaWiFS remote sensing data. Slope and Intercept are the linear regression results from the satellite retrievals *vs.* the *in situ* ones.

Target	Count	Slope	Intercept	*R*^2^	*p*	RMSE/mg/m^3^
Annual	58	0.67	0.71	0.57	0.0001	2.32
Before DINEOF	40	0.79	0.36	0.61	0.0001	1.78
After DINEOF	18	0.56	1.26	0.54	0.0001	2.53
Spring	13	0.71	0.90	0.81	0.0001	2.44
Summer	21	0.36	0.97	0.43	0.0010	2.62
Autumn	18	2.02	−0.39	0.90	0.0001	2.23
Winter	6	1.28	−0.14	0.59	0.0740	0.61

### 3.2. Spatial and Temporal Variations of Chl-a Concentration

The spatial distribution of the average Chl-a concentrations from 1998 to 2010 is described in Figure 1. The Chl-a concentration decreases from the coastline to the open sea. A tongue-shaped high value area near the Yangtze River estuary extends into the open sea. This is known as the Yangtze River Plume, due to terrigenous runoff water mixing with seawater [37]. Because of the effect of the Taiwan Warm Current, Coriolis and southwest monsoons, the Yangtze River Plume extends northeastward [38]. Hypersaline diluted water usually floats within 10 m of the surface and an intense halocline exists underneath [4]. In summer, the temperature of the runoff is higher than that of the seawater. When the runoff reaches the sea, accompanied by a significant vertical change of water temperature, an epilimnion occurs. The epilimnion is beneficial to the stability of seawater. Under these stable conditions, the residence time of phytoplankton in the mixed layer depth is lengthened, and, combined with the rich soluble nutrients of runoff, phytoplankton growth is favored [39].

**Figure 4:**
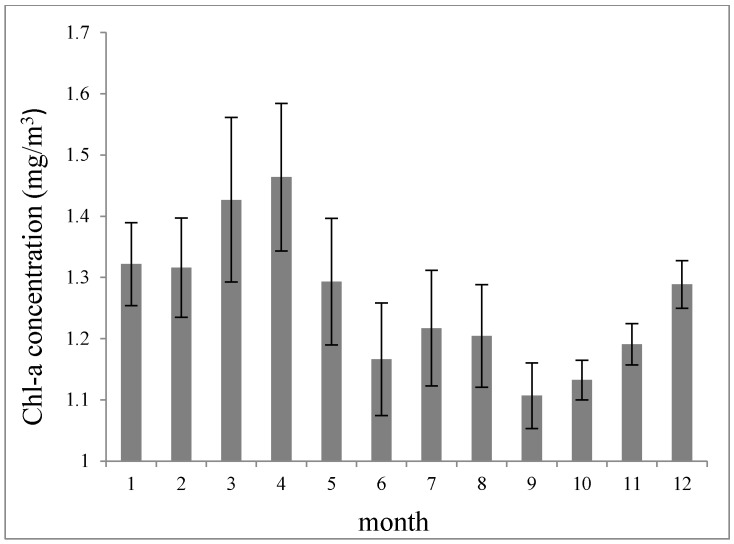
Monthly average variation trend of Chl-a concentration. Error bars show the standard deviation intervals of the mean.

Figure 4 shows the monthly average variations of Chl-a concentration and the standard deviation intervals from September 1997 to December 2010. Peaks appear in March and April (Chl-a > 1.4 mg/m^3^), and then drop when summer approaches. The lowest Chl-a values appear in September (Chl-a = 1.1 mg/m^3^), and then increases with the approaching winter, but there is a small rise in July and August, creating a bimodal seasonal behavior. Similar phenomena were also found in a study by Cong *et al*. [40] near the Yangtze River Estuary. The small rise of Chl-a in July and August is generated by the terrigenous water which brings with it abundant nutrients. The reason will be discussed in the next section. The standard deviation is largely consistent with the monthly values. Both the average value and standard deviation of Chl-a concentrations are high in spring, and low in autumn. This means that there is a high variability in the bloom phenology and among the years.

### 3.3. Spatial Distribution of the Correlation Coefficient of Chl-a Concentration and Yangtze River Discharge

**Figure 5:**
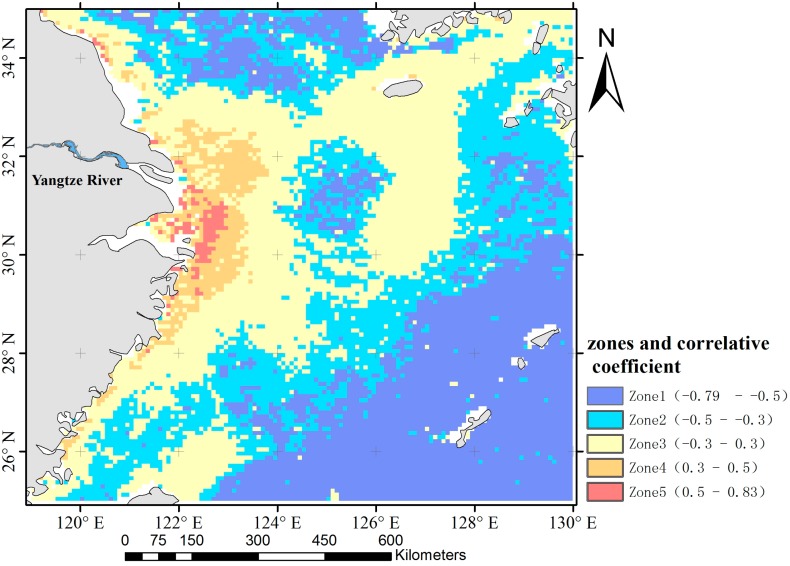
Distribution of correlation coefficient of the monthly average discharge and monthly average SeaWiFS-retrieved Chl-a.

To explain the impact of terrigenous water on Chl-a concentrations in the East China Sea, a correlation analysis is calculated between the discharge and Chl-a (Figure 5). The correlation coefficient (r) is positive in the area around the estuary (from 0.3 to 0.83), while it is negative further away from the estuary (from −0.3 to −0.79). According to the classification method of Huang *et al.* [41]: |*r*| < 0.3 indicates uncorrelated, 0.3 < |*r*| < 0.5 indicates low correlated, 0.5 < |*r|* < 0.8 indicates medium correlated, 0.8 < |r| < 1.0 indicates strongly correlated, the research area is separated into five zones according to the correlation coefficients as shown in the legend in Figure 5. The areas of positive medium correlation and strongly correlated (0.5 < r < 0.83) are combined, because only a small number of pixels are contained in these area. The zones were named Zone 1 to Zone 5 (the correlation coefficient ranging from negative to positive). Meanwhile, the zoning method considers the spatial characteristic of Chl-a concentration and the range of influence from the terrigenous water in the study area. Therefore, it is suitable for the research purpose. Zone 1 and Zone 2 show a negative correlation. They are far from the coast and therefore the Chl-a is not affected by land. Zone 4 and Zone 5 show a positive correlation. They are near the coast and therefore the Chl-a is influenced by land. Zone 3 is uncorrelated and is the transition zone between the previous two situations. 

**Figure 6:**
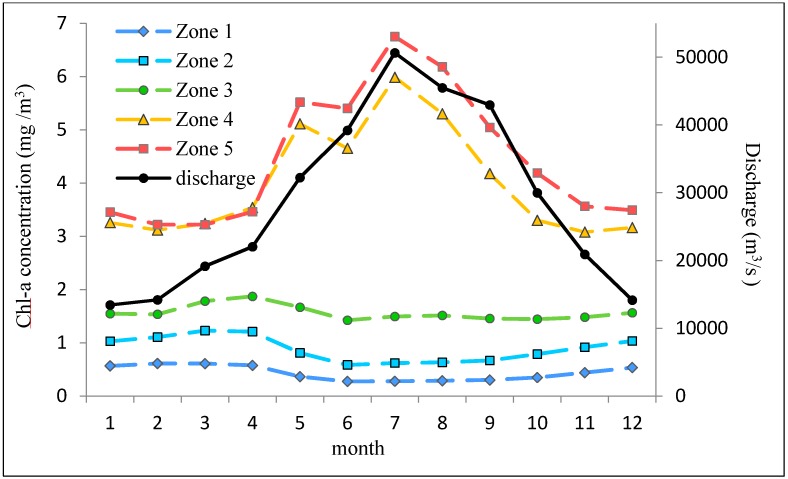
Monthly average of Chl-a concentration in each zone and discharge observed at the Datong hydrological gauging station.

Figure 6 shows the statistical analysis of the monthly average Chl-a variation from September 1997 to December 2010. The Chl-a concentration in Zone 4 and Zone 5 is much higher than that in the other zones. The annual variation pattern for Zone 1 to Zone 3 shows Chl-a concentration to be high in winter and spring, with a peak in April and low in summer and autumn. The trend is similar to that in the entire area shown in Figure 4. Zone 4 and Zone 5 show similar trends but are quite different from the other three zones. In Zone 4 and Zone 5, Chl-a concentrations jump to a high from May to August, with a peak appearing in July and a low in winter. This trend matches well with the seasonal variation of the Yangtze River discharge.

Diluted water from the Yangtze River is distributed in the estuary area where salinity is < 30~32 psu [24], and seasonal behavior is controlled by the current system and wind condition (summer monsoon) [15,17]. The diluted water, affected by current and wind, extends northeastward and turns into the plume mentioned in Section 3.2. Therefore discharge from the Yangtze River could not extend to the south of the Yellow Sea and southeast of the East China Sea. This means the highly negative correlation coefficient in these areas could not be due to the contributing of discharge to the Chl-a.

Different zones show different patterns of Chl-a annual variation. Therefore, the bimodal phenomenon is the result of an overly large target study area. By zoning and analyzing the adjacent sea area of the Yangtze River estuary separately, the inshore and open sea areas show quite diverse pattern of annual variations.

Near the coast, the Chl-a concentration is high in spring and summer, but low in autumn and winter. This coincides with the research of Zhou *et al.* [42]. With abundant nutrients brought by terrigenous water the water temperature rises in spring and summer, making it suitable for phytoplankton growth, so Chl-a concentration is high. In the autumn and winter the water temperature decreases with the prevailing northeasterly wind, the intensified convection sends the nutrients up to the surface leading to the lack of illumination. Limited by the low temperature and insufficiency of illumination, biomass and Chl-a concentration both decrease [6]. 

With large amounts of industrial and agricultural wastes and domestic sewages carried by the Yangtze River Discharge, and also the high-intensive aquaculture activities in the coastal waters, combining with the optimum temperature in spring, the Chl-a increased and accumulated fast, and then caused a red tide disaster. May is the most likely time for a harmful algae bloom [42]. Zone 4 and Zone 5 are the areas where blooms mostly occur [43]. The seasonal behavior of Zone 4 and Zone 5 are derived from the average values during 13 years, including the years with and without blooms. The bloom years raise the average Chl-a in May, Therefore, it is a high risk of red tide disaster in May, while April and June had similar cases. The bloom will pollute the water quality and destroy the environment in the coastal waters [44]. Phytoplankton, fish and shellfish are killed by algae blooms and accumulated toxins. The toxins threaten human health and life safety throughout the food chain.

The temperature is low in winter in the open sea. Therefore, with sufficient vertical mixing and abundant nutrient substances, Chl-a concentration is high. To the east of the East China Sea, the Chl-a concentration declines in spring due to the reduction of vertical mixing. If a line is drawn between Taiwan and Kyushu Island, the Chl-a concentration in the east is particularly low. Chl-a concentration is lowest in the summer because of the limited seawater stratification. Towards the end of autumn the temperature gradually declines, and with sufficient vertical mixing, Chl-a rises in the open sea [45].

It should be noted that the correlation coefficient between discharge and Chl-a appears negative in the area near the location of 125.5° E, 31.5° N. This is because the area is located within the transition zone and is under the strong predominant influence of discharge and the predominant influence of environmental factors on Chl-a concentration. The baseline of Chl-a concentration in this area is relatively high in spring, but its value decreases because of consumption by zooplankton, and reaches the lowest at the end of spring [46]. Although the amount of discharge is high in summer and brings substantial quantities of nutrients into this area, the replenishment is insufficient to counteract the consumption by zooplankton. Thus, the seasonal variation of Chl-a appears high in spring and low in summer, while the discharge is low in spring and high in summer. This directly results in the negative correlation coefficients between discharge and Chl-a.

### 3.4. Analysis of the Time Series Characteristics of Chl-a Concentration and Yangtze River Discharge

Zoning analysis shows the different spatial patterns of Chl-a concentration. The sinusoidal curve model is used to represent the evolving monthly variation of Chl-a concentration in each zone, based on Equation (1) with MATLAB. The curve fitting parameters are shown in Table 2.

**Table 2:** Fitted parameters of sinusoidal models in various zones and Yangtze River discharge. B/A, the rate of change; *R*^2^, the model coefficient of determination; Significance, Significance level ($\left|\frac{B}{\sigma_{B}}\right|$).

Target	A	B	C	D	E	B/A	*R*^2^	Significance
Zone 1	0.41	0.0003	0.14	12.05	0.77	0.0006	0.93	5.72
Zone 2	0.85	0.0004	0.22	12.08	0.79	0.0005	0.90	4.83
Zone 3	1.52	0.0005	0.12	12.22	0.62	0.0003	0.42	2.79
Zone 4	3.82	0.0023	0.93	11.94	−2.19	0.0006	0.73	3.19
Zone 5	4.30	0.0020	1.18	11.98	−2.28	0.0005	0.79	2.51
Discharge	35616.21	−115.79	13358.16	11.92	−2.48	-0.0033	0.83	7.19

The model coefficient of determinations (*R*^2^) in Zone 1 and Zone 2 are high (*R*^2^ >0.9). Chl-a seasonal behavior in the open sea is mainly controlled by meteorological factors, such as photo-synthetically active radiation, sea surface temperature and sea surface wind speed [47,48]. The stable control factors mean that the variation pattern corresponds well to the sine function shape. *R*^2^ in Zone 4 and Zone 5 is also high (*R*^2^ > 0.7), because the two zones are located on the coast. Except for meteorological factors, they are mainly controlled by the discharge from the Yangtze River. These control factors are seasonally stable, too. *R*^2^ is low in Zone 3 (*R*^2^ > 0.4) because the Chl-a concentration is affected by meteorological factors and the discharge from the Yangtze River at the same time. However, the two factors have different seasonal variations. Zone 3 is the transitional zone from coast to open sea and the control factors are complicated. Yangtze River discharge corresponds well with the sine function with stable periodic changes (*R*^2^ = 0.83).

The characteristic of each zone is reflected differently by each parameter of the model. ($\left|\frac{B}{\sigma_{B}}\right|$) indicates the significance level of the model in a linear relationship. $\left|\frac{B}{\sigma_{B}}\right|>2$ in the models of Chl-a in every zone and Yangtze River discharge, so we can consider that the monthly average of Chl-a concentration and Yangtze River discharge variation trends are notable when the confidence level is 95%.

The magnitude (A) of Chl-a concentration demonstrates that there is a generally diminishing trend from offshore to open sea. 

The increment (B) and growth rate (B/A) in all zones are small, but Chl-a concentration shows a slight growth trend with discharge being slightly reduced. Because of the filling of the Three Gorges Dam, there are reductions of onshore freshwater [40]. However, Yamaguchi *et al.* [49] suggested that the changes in the Yangtze River Discharge have not directly affected the increase of Chl-a concentration. The gradual increase in Chl-a concentration is indicative of eutrophication and might be caused by the increase and accumulation of nutrients from the Yangtze River and other sources including other rivers, atmospheric deposition and aquatic feed. These may be the reason for red tide disaster and marine pollution. As reported, the occurrence of harmful algal blooms in the Yangtze River Estuary has been increasing [21]. Anthropogenic activities have a great impact upon water quality in coastal environments.

The amplitude of variation (C) indicates that the trend for Chl-a concentration to diminish is in accordance with the distances from the coastline, while Zone 3 appears low. The main influencing factor is fresh water from the Yangtze River and the accompanying nutrients from the land. 

The change cycle (D) is about 12 months, indicating an obvious cyclical annual change of Chl-a concentration and Yangtze River discharge. 

According to Equation (6), the initial phase (E) shows the time difference of wave pattern between discharge and each zone (Differ). The Differ between discharge and every zone is shown in Table 3. The time differences between the five zones and the discharge are greater for larger distances from the coast, which indicates that the influence of discharge on Chl-a decreases gradually from coast to open sea.

**Table 3:** The time difference of wave pattern between discharge and each zone (Differ).

Zone	Zone 1	Zone 2	Zone 3	Zone 4	Zone 5
Differ/Month	6.21	6.25	5.92	0.56	0.38

### 3.5. Temporal Variations of Chl-a Concentration from the Influence of Discharge and the Transport Process of Diluted Water from the Yangtze River

The diluted water spreads eastward in the East China Sea to Jeju Island with a time lag of 1 to 2 months after the discharge [24]. Therefore the present study strives to find the period when Chl-a concentration is influenced by the discharge. Following the result acquired by Kim *et al.* [24], we chose the research area around Jeju Island indicated in Figure 1. The Chl-a concentrations of every month from this area, are compared to the discharge with and without time lag. Table 4 shows the correlation coefficient of discharge and Chl-a. The high correlation (correlation is significant at the 0.05 level or 0.01 level) appears from July to November. The discharge in July has the highest correlation with the Chl-a in September (*r* = 0.859), the discharge in August has the highest correlation with Chl-a in September (*r* = 0.847), and the discharge in September has the highest correlation with Chl-a in November (*r* = 0.783). Therefore the result (*r*) indicates that the Chl-a between September to November is controlled by the discharge between July to September with a lag of 1 to 2 months.

**Table 4:** Correlation coefficients between Chl-a concentration and amount of the summer Yangtze discharge from 1998 to 2007 with and without time lag.

Target		Discharge											
		1	2	3	4	5	6	7	8	9	10	11	12
**Chl-a concentration**	**1**	0.371											
	**2**	−0.112	−0.280										
	**3**	0.108	−0.074	0.212									
	**4**	−0.331	−0.432	−0.119	0.002								
	**5**	−0.148	−0.101	0.092	−0.238	−0.714 ^*^							
	**6**	0.126	0.138	0.280	0.243	−0.375	0.223						
	**7**	−0.284	−0.010	−0.022	−0.146	0.034	0.088	−0.508					
	**8**	0.175	0.220	0.213	−0.300	−0.516	−0.439	−0.371	0.091				
	**9**	0.452	0.205	0.367	0.268	−0.086	0.290	0.859 ^**^	0.847 ^**^	0.740 ^*^			
	**10**	−0.001	−0.163	−0.044	0.013	0.341	0.386	0.793 ^**^	0.745 ^*^	0.617	0.233		
	**11**	0.636	0.569	0.341	0.339	0.032	0.416	0.562	0.723 ^*^	0.783 ^**^	0.625	0.169	
	**12**	0.308	0.186	0.302	0.362	0.035	0.034	0.240	−0.031	0.118	0.460	0.384	0.239

^**^, Correlation is significant at the 0.01 level; ^*^, Correlation is significant at the 0.05 level.

**Figure 7:**
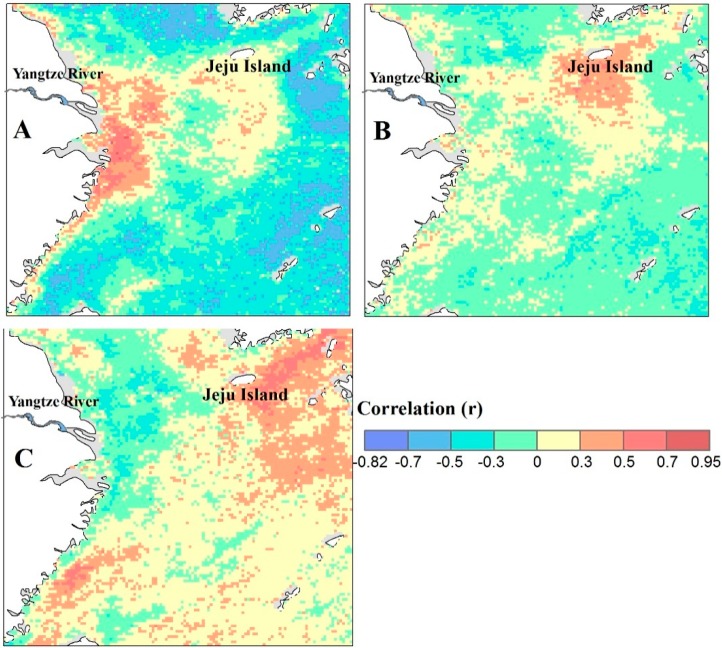
Correlation coefficients (r) between Chl-a concentration and discharge from July to November. Chl-a concentration had a time lag of 0 (**A**); 1 (**B**); and 2 (**C**) months after the discharge.

This paper also analyzes the transport process of Yangtze River diluted water. We calculate the correlation (r) between Chl-a concentration and discharge from July to November with a time lag of 0 (A), 1 (B), and 2 (C) months (Figure 7). The area with the highest positive correlation moves from the Yangtze River mouth to the east of Jeju Island with lags of 0–2 months, and the time lags are longer with areas far from the Yangtze River mouth. This result suggests that the area of Yangtze River diluted water does indeed relate to discharge as reported by Lie *et al.* [15] and Kim *et al.* [24]. It also shows that Yangtze River diluted water in summer extends from inshore of the Yangtze River mouth to the east of Jeju Island, and the summer diluted water influences the growth of Chl-a where it extends to within two months. In view of the above studies, the nutrition and pollutants may get to the open sea and affect the seawater quality along with the transference of Yangtze River diluted water.

## 4. Conclusions

Based on 13 years of remotely-sensed Chl-a data from SeaWiFS, the spatial and temporal variations of Chl-a and the influence of Yangtze River discharge on its concentration have been studied. The results revealed that the gradient pattern of Chl-a concentration are that a high concentration is near the coast and a low one is in the open sea as the terrigenous runoff which provides abundant nutrients for phytoplankton near the coast. The temporal pattern, in the coastal areas, Chl-a concentration is high in late spring and summer while low in winter, which is mainly influenced by the discharge which brings plenty of nutrients. In the open sea areas, Chl-a is high in early spring and low in summer, mainly driven by meteorological factors. The impact of Yangtze River Discharge on Chl-a concentration reduces gradually from inshore to open sea.

Indicated by the spatial and temporal variations of satellite-derived Chl-a, a red tide disaster is most likely to occur in May in the coastal areas of Yangtze River Estuary. Furthermore, the gradual increase in Chl-a concentration is indicative of eutrophication, which will influence the biogeochemical dynamics of the ocean ecosystem and might bring more damages in the future as a result of the feedback of increasing impact of human activities on the marine environment. This is a potential risk to the human living environment and health to some extent.

The Chl-a data between September to November shows an obvious response to the discharge between July to September with a lag of 1 to 2 months. The main pathway for the Yangtze River diluted water in summer is from inshore of the Yangtze River mouth to the east of Jeju Island and it has a significant effect on Chl-a. The transference may take the nutrition and pollutions from the coastal area to the open sea, which will increase the risk of diffusion of red tide disasters to the open sea.

The results of this study are typical for a large delta area with plentiful terrigenous water flowing into the broad ocean, and we focus here on the impact of discharge on the Chl-a concentration. In further study, other factors such as temperature, ocean current, and seawater salinity should be taken into account.
